# Brain NAD Is Associated With ATP Energy Production and Membrane Phospholipid Turnover in Humans

**DOI:** 10.3389/fnagi.2020.609517

**Published:** 2020-12-16

**Authors:** Bernard Cuenoud, Özlem Ipek, Maya Shevlyakova, Maurice Beaumont, Stephen C. Cunnane, Rolf Gruetter, Lijing Xin

**Affiliations:** ^1^Nestlé Health Science, Lausanne, Switzerland; ^2^School of Biomedical Imaging & Imaging Sciences, King's College London, London, United Kingdom; ^3^Clinical Development Unit, Nestlé Research Center, Lausanne, Switzerland; ^4^Department of Medicine, Université de Sherbrooke and Research Center on Aging, Sherbrooke, QC, Canada; ^5^Laboratory for Functional and Metabolic Imaging, Ecole Polytechnique Fédérale de Lausanne (EPFL), Lausanne, Switzerland; ^6^Center for Biomedical Imaging, Ecole Polytechnique Fédérale de Lausanne, Lausanne, Switzerland

**Keywords:** NAD, brain energy, ATP metabolism, ^31^P-MRS, redox ratio, membrane phospholipid metabolism, creatine kinase, ATP synthase

## Abstract

The brain requires a large amount of energy, mostly derived from the metabolism of glucose, which decreases substantially with age and neurological diseases. While mounting evidence in model organisms illustrates the central role of brain nicotinamide adenine dinucleotide (NAD) for maintaining energy homeostasis, similar data are sparse in humans. This study explores the correlations between brain NAD, energy production and membrane phospholipid metabolism by 31-phosphorous magnetic resonance spectroscopy (^31^P-MRS) across 50 healthy participants including a young (mean age 27.1-year-old) and middle-aged (mean age 56.4-year-old) group. The analysis revealed that brain NAD level and NAD^+^/NADH redox ratio were positively associated with ATP level and the rate of energy production, respectively. Moreover, a metabolic network linking NAD with membrane phospholipid metabolism, energy production, and aging was identified. An inverted trend between age and NAD level was detected. These results pave the way for the use of ^31^P-MRS as a powerful non-invasive tool to support the development of new therapeutic interventions targeting NAD associated phospho-metabolic pathways in brain aging and neurological diseases.

## Introduction

Nicotinamide adenine dinucleotide (NAD) is a vital cofactor involved in brain bioenergetics for metabolism and ATP production, the energy currency of the brain (Lautrup et al., 2019). NAD exists in an oxidized (NAD^+^) or reduced (NADH) form, with NAD^+^/NADH (the redox ratio) being an important determinant of cytosolic and mitochondrial metabolic homeostasis. NAD^+^ plays an essential role in glycolysis and the citric acid (TCA) cycle, by its ability to accept hydride equivalents, forming NADH during adenosine triphosphate (ATP) production. NADH is one of the central electron donors in oxidative phosphorylation in the mitochondria, providing electrons to the electron transport chain (ETC) to generate most of the ATP. These reactions support the high energy demands of the brain, particularly neurons, which are derived mostly from glucose metabolism under physiological conditions (Dienel, 2019). Additionally, NAD^+^ is a key substrate for multiple NAD^+^-dependent enzymes implicated in neuronal integrity. It is consumed by at least four classes of enzymes involved in genomic stability, mitochondrial homeostasis, adaptive stress responses, and cell survival (Katsyuba et al., 2020). Emerging evidence also implicates NADH directly in the microglial immune response induced by changes in brain energy metabolism, possibly via binding and activation of the transcriptional co-repressor C-terminal binding protein CtBP (Shen et al., 2017).

While mounting evidence in pre-clinical models illustrates the central role of NAD for neuronal homeostasis, available data for the human brain are sparse (Gilmour et al., 2020; Katsyuba et al., 2020). Recently, quantitative and non-invasive measurement of NAD^+^ and NADH in human brain was demonstrated using 31-phosphorus magnetic resonance spectroscopy (^31^P-MRS) at high magnetic field (Zhu et al., 2015). This technique provides a unique non-invasive tool to monitor NAD level and NAD^+^/NADH redox ratio changes in humans under healthy and pathological conditions (Zhu and Chen, 2018; Downes et al., 2019).

Based on extensive *in-vitro* and animal data, one would predict that aging and many neurological diseases would lead to a decrease in brain total NAD (tNAD, NADH+NAD^+^) levels, NAD^+^/NADH redox ratio, or both (Verdin, 2015; Lautrup et al., 2019; Katsyuba et al., 2020; McReynolds et al., 2020). In the first human study investigating the effect of aging on brain NAD, it was found that tNAD, NAD^+^, and NAD^+^/NADH redox ratio were indeed all decreased with age while NADH increased (Zhu et al., 2015). In psychotic disorders such as schizophrenia and bipolar disorders, an increase in NADH and a decrease in NAD^+^/NADH redox ratio was consistently observed in the frontal lobe of the brain vs. age-matched controls (Chouinard et al., 2017; Kim et al., 2017), but no change in NAD^+^ level was detected. In other prevalent neurodegenerative disorders such as mild cognitive impairment or Alzheimer's disease, where reduced brain glucose metabolism has been shown to precede the onset of cognitive symptoms (Cunnane et al., 2020), further investigations are needed to determine brain NAD status.

Therapeutic interventions aimed at improving brain NAD homeostasis in aging and neurodegenerative diseases have been a major focus in recent years, with many encouraging pre-clinical outcomes (Rajman et al., 2018; Lautrup et al., 2019; Katsyuba et al., 2020; McReynolds et al., 2020), but clear clinical benefits are yet to be reported in humans. Recently, we found that a nutritional ketogenic intervention was able to increase brain NAD^+^ level and to increase the brain NAD^+^/NADH redox ratio in healthy participants (Xin et al., 2018), providing the first indication in humans that an alternative brain energy substrate such as ketones could modulate brain NAD status.

Aside from NAD^+^ and NADH, ^31^P-MRS can simultaneously measure other phospho-metabolites involved in energy and membrane phospholipid metabolism (Zhu and Chen, 2018; Downes et al., 2019), allowing the investigation of their association with the NAD status. This should provide critical mechanistic information such as how the impact of NAD redox dysregulation in aging and neurological diseases affects the brain levels of ATP and phosphocreatine (PCr). Furthermore, it should also shed light on the possible role of NAD for phospholipid status in neuronal membranes. In addition to these static measurements, ^31^P-MRS can quantify the global cerebral energy demand by measuring the kinetic rate of brain energy enzymes, i.e., ATP synthase and creatine kinase (Lei et al., 2003). These values, determined by ^31^P magnetization transfer, provide the possibility of determining how ATP production rate is related to the NAD^+^/NADH redox status in the human brain.

Here we seek to establish the links between NAD, energy production and membrane phospholipid metabolism in the healthy human brain, and to determine how these associations are influenced by age. The use of a high magnet field strength (7 Tesla) provides high spectral sensitivity and signal dispersion that permits the assessment of individual phospho-metabolites and energy enzymatic rates. This study provides the first integrated phospho-metabolic network of the human brain across 50 healthy participants, highlighting significant associations between NAD and ATP energy production and phospholipid membrane metabolism. This paves the way for the use of ^31^P-MRS as a powerful non-invasive method to support the development of new therapeutic interventions targeting NAD associated phospho-metabolic pathways in brain aging and neurological diseases.

## Materials and Methods

### Study Participants and ^31^P-MRS Measurement of the Human Brain

A total of fifty healthy participants were included in the analysis. They were separated into a young group (*n* = 25, mean age 27.1 ± 6.1 standard deviation) and a middle-aged group (*n* = 25, mean age = 56.4 ± 6.4), with an overall age distribution between 19 and 74 years old and a male/female ratio of 31/19. See Supplementary Material for participant demographic details (Supplementary Table 1) and age distribution (Supplementary Figure 1). ^31^P-MRS data were derived from a group of middle-aged healthy participants acquired as part of a larger study and were combined with data from the young group obtained in our recent study (Xin et al., 2018). None of the present data have been previously published. Each scan was recorded in the fasted state between 8 and 10 a.m., and each participant was asked not to eat after 8 p.m. the evening before and not to undertake strenuous exercise the day before the scan. All participants provided written informed consent under the approval of the Swiss cantonal ethics committee.

MR experiments were performed on a 7 Tesla/68 cm MR scanner (Siemens Medical Solutions, Erlangen, Germany) with an in-house-built ^1^H quadrature surface coil (10 cm-diameter) and a single-loop ^31^P coil (7 cm-diameter) for the occipital lobe. Radiofrequency tissue heating parameters were simulated by the finite-difference time-domain method in Sim4Life (ZMT, Zurich Switzerland). B_0_ field inhomogeneity was optimized in a VOI (50 × 30 × 40 mm^3^) using first- and second-order shimming with FAST(EST)MAP (Gruetter, 1993). The flip angle of the RF pulse was calibrated prior to acquisition using the PCr signal.

All ^31^P resonance signals including NAD redox metabolites, NAD^+^ and NADH, energy related metabolites [PCr, α-ATP, β-ATP, γ-ATP, intracellular/extracellular inorganic phosphate Pi_int_ and Pi_ext_, UPDG (uridine diphosphoglucose)], and membrane related metabolites [PE (phophoethanolamine), PC (phosphocholine), GPC (glycerophosphocholine), GPE (glycerophosphoethanolamine), MP (membrane phospholipid)] were measured using a pulse acquire sequence with the following parameters: 200 μs hard pulse, spectral bandwidth = 6,000 Hz, 2,048 data points, TR = 3 s, average = 320 (young group) or 512 (middle-age group).

Forward rate constants of creatine kinase (k_CK_) and ATP synthase (k_ATP_) were measured by ^31^P saturation transfer experiments (Xin et al., 2018) with the following three measurements (saturation time τ_sat_ of 8.25 ms, TR = 16 s, average = 32): (1) steady-state saturation measurement for γ-ATP, saturation pulses at −2.5 ppm; (2) control measurement for Pi, saturation pulses at 12.2 ppm; (3) control measurement for PCr, saturation pulses at 2.5 ppm. The BISTRO (B_1_-insensitive selective train to obliterate signal) scheme (Luo et al., 2001; Xin et al., 2018) was applied for saturation prior to a pulse acquire sequence.

### ^31^P MRS Quantification

^31^P MR spectra were summed after frequency and phase correction and then analyzed by LCModel using a basis-set composed of simulated ^31^P spectra of PCr, α-ATP, β-ATP, γ-ATP, Pi_int_, Pi_ext_, PE, PC, GPC, GPE, MP, NADH, NAD^+^, UPDG with their respective linewidths (Xin et al., 2018). Values for each metabolite was expressed as a percent of the total ^31^P signal (sum of PCr, α-ATP, β-ATP, γ-ATP, Piint, Piext, PE, PC, GPC, GPE, MP, NADH, NAD^+^, and UPDG signals).

The rate constants, k_CK_ and k_ATP_, were calculated from steady-state saturation experiments using the following equation: Mss = Mc/(1+k·T_1_^int^), assuming intrinsic T_1_ of PCr and Pi at 7T are 4.9 and 3.8 s (Du et al., 2007). Mss and Mc are signal intensities of Pi or PCr obtained respectively from steady-state and control measurements.

### Data Analysis and Statistical Methods

Fourteen parameters were included in the analysis: age, NAD^+^, NADH, tNAD (the sum of NAD+ and NADH), NAD^+^/NADH, ATP (as the average of α-ATP, β-ATP, and γ-ATP), PCr, Pi (as Pi_int_), k_CK_, k_ATP_, PE, PC, GPC, and GPE.

Percent differences in the parameters between the young and middle-aged groups were calculated as the difference of the mean between middle-aged and young participants divided by the mean value for the young group expressed as percent (**Figure 2**). Significance was determined using unequal variances *t*-tests between the two groups.

Spearman's rank correlation test was carried out in Matlab (R2018b, The MathWorks, Inc.) and was used to evaluate the association between variables. *P*-values and Spearman's rank correlation coefficient, *rho*, were determined (**Figure 3**). To visualize the results in the context of the brain energy metabolism and structure, the variables were grouped according to their relevance to energy metabolism, NAD, and phospholipid metabolism, and their association depicted by a line, the width of which corresponds to the strength of the correlation (**Figure 4**).

To estimate the impact of potential false positive results in the context of multiple testing, *p*-values were controlled by the False Discovery Rate (FDR) procedure with *q* = 0.05 (Benjamini and Hochberg, 1995), providing a solid basis for drawing conclusions about statistical significance. This was used for both the comparison between the older and young group and the Spearman's rank correlation association and is fully reported in Supplementary Tables 2, 3.

## Results

### ^31^P MR Spectra in Human Occipital Lobe

**Figure 1:**
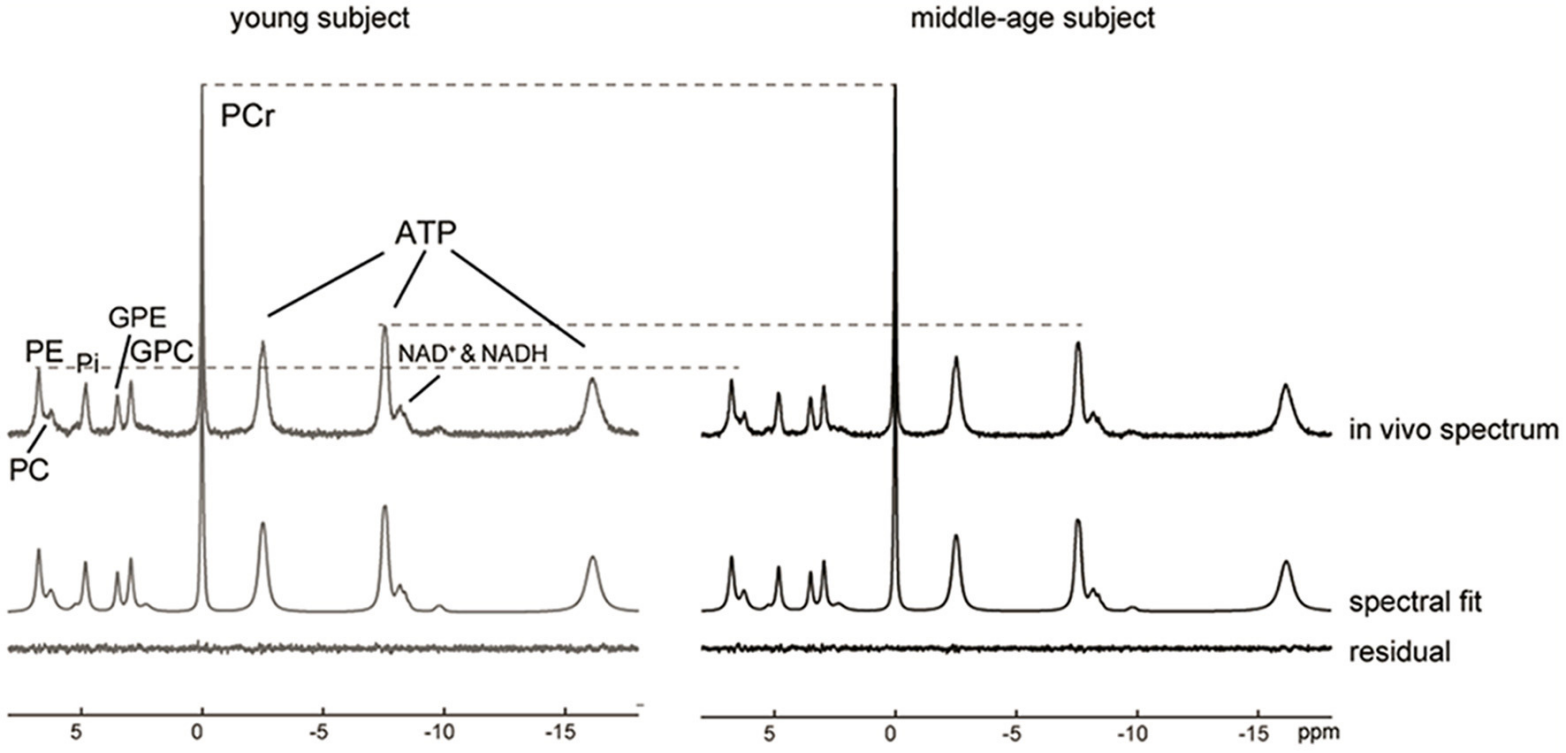
^31^P MR spectra, LCModel spectral fits and fit residuals in the occipital lobe of a young and a middle-aged participant. No apodization was applied. For visual comparison, the baseline was removed by subtraction of the spline fit and the two spectra were then normalized to PCr peak height. A reduction in the ATP and PE peaks was observed in the middle-aged subject as indicated by the dashed lines.

Representative ^31^P MR spectra acquired for the occipital lobe of a young and a middle-age subject exhibited excellent spectral resolution at 7 Tesla that allowed the detection of 13 resonances including separate detection of NAD^+^, NADH, GPE, GPC, PE and PC (Figure 1). After normalizing the two spectra to the PCr peak, lower ATP and PE signals could be visually observed in the middle-aged participant's spectrum.

### Significant Differences Between the Middle-Age and Young Groups

**Figure 2:**
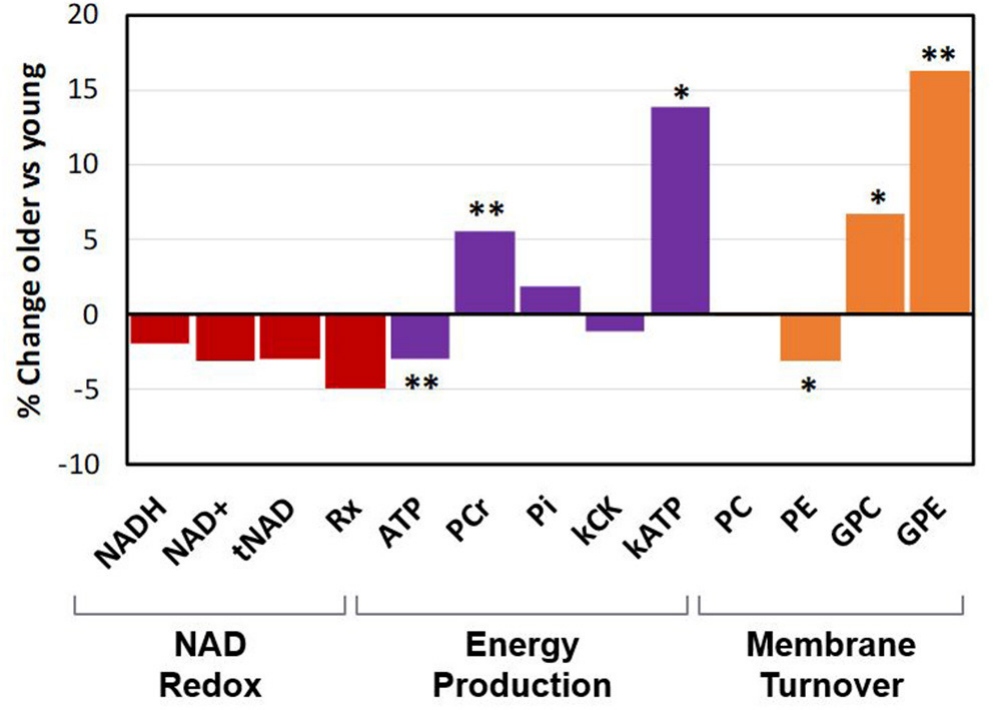
Percent change in phospho-metabolites and in metabolic rate constants between the middle-aged group vs. the young group (*p < 0.05; **p < 0.01). Rx is NAD+/NADH redox ratio.

To determine how age impacts parameters related to NAD, energy production and phospholipid membrane metabolism, differences between the middle-age and the young group were measured. A mean difference of 29 years (*p* < 0.01) separated the two age groups. Six parameters were statistically different between the two groups (Figure 2): as age increased, there was an increase in PCr (+5.6%), GPC (+6.8%), and GPE (+16.2%) levels, and k_ATP_ (+13.9%), together with a significant decrease in ATP (−3.0%) and PE (−3.1%) levels. Difference in ATP, PCr, GPE, and GPC remained statistically significant after FDR correction. The mean values for k_ATP_ for the middle-age and young groups were 0.149 ± 0.023 s^−1^ and 0.131 ± 0.029 s^−1^, respectively; and those for k_CK_ were 0.228 ± 0.027, and 0.230 ± 0.022 s^−1^, (*p*-values middle-age vs. young for k_ATP_ and k_CK_ were 0.022 and 0.713). The NAD related values all decreased by 2–5% with increasing age but did not reach statistical significance (*p* > 0.1).

### Correlations in Brain Metabolic Network

**Figure 3:**
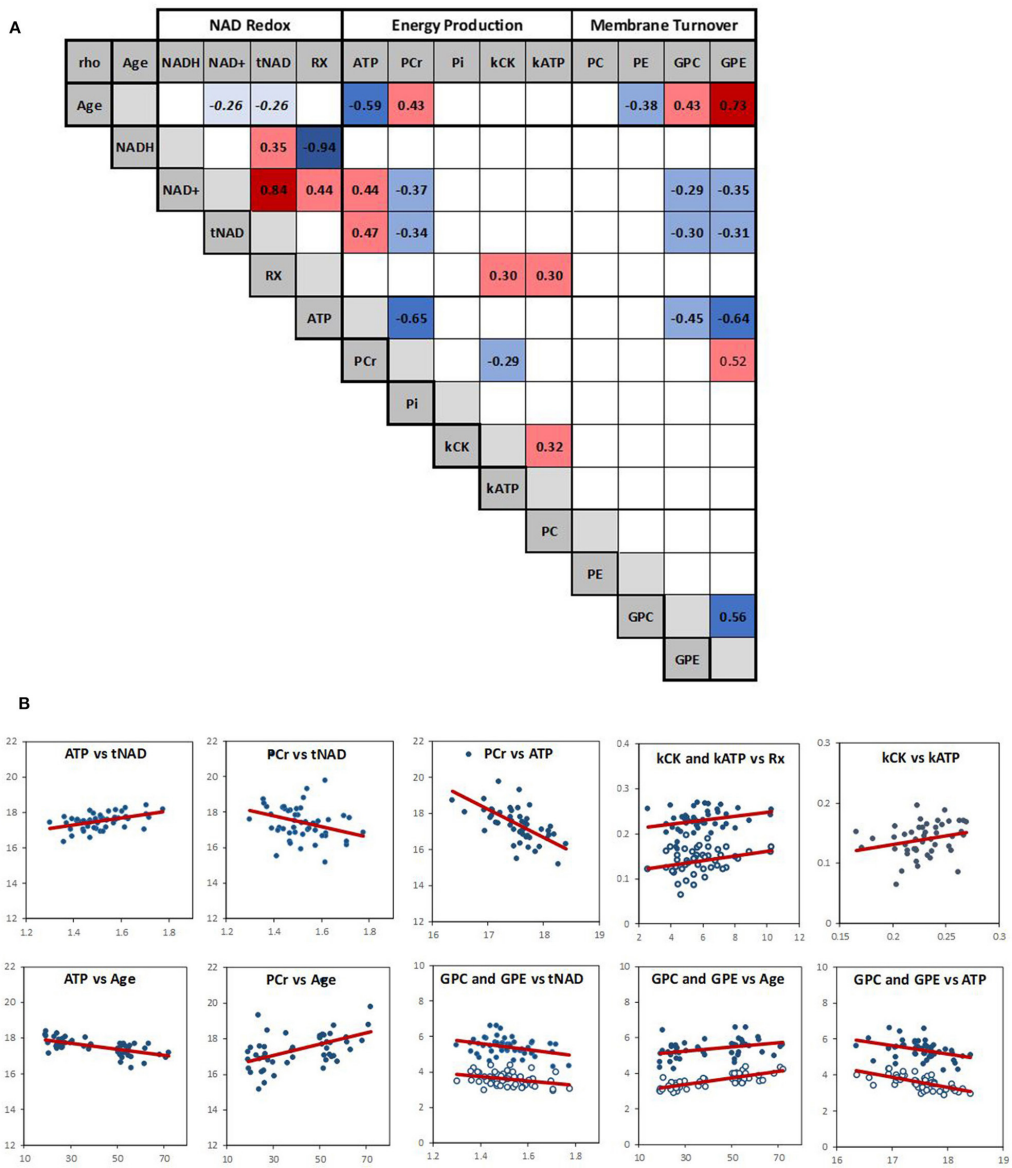
**(A)** Spearman correlation matrix across the 14 variables (RX is NAD^+^/NADH redox ratio). Numbers are rho values, and color coding indicate a significant (*p* < 0.05) positive (red) or negative (blue) correlation. Color shading indicate strength of the correlation. There was a negative trend (*p* ~ 0.06) between age and NAD^+^ and tNAD. Empty squares indicate no significant correlation. **(B)** Scatter plots of the main correlations with red linear regression line; open circle: k_ATP_ and GPE.

**Figure 4:**
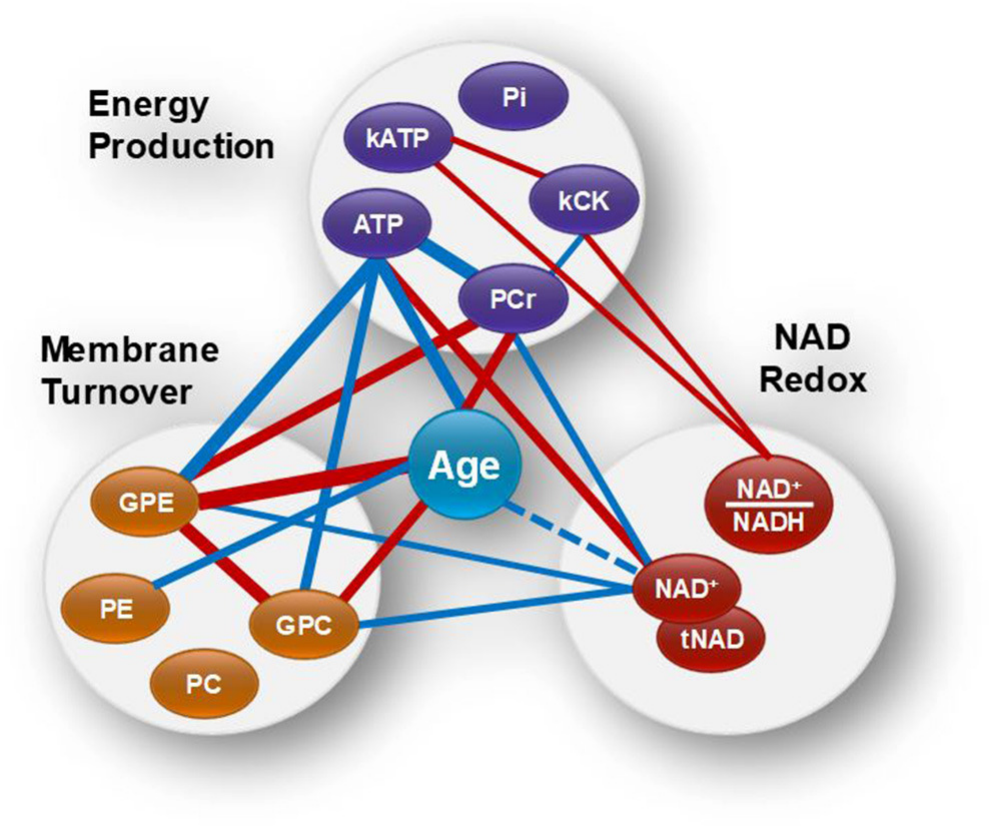
Brain age and the networks between NAD status, energy production and membrane phospholipid metabolism. Red and blue lines indicate positive and negative correlations, respectively. Wider lines correspond to a stronger correlation. NAD^+^ and tNAD had similar network associations.

To investigate simultaneously the dependence between the multiple variables, a Spearman correlation matrix was established. Twenty six moderate-to-strong (Rho > 0.29) significant correlations were found out of a possible total of 91 (Figure 3A). Fifteen correlations passed the FDR correction for multiplicity (Supplementary Table 3). Scatter plots of the main correlation are represented (Figure 3B). To best visualize how the NAD redox, energy production and membrane metabolism variables interacted together and with age, a network between NAD status, energy production and membrane phospholipid metabolism and age was built (Figure 4).

Within the energy and redox networks, tNAD-ATP, and PCr-ATP were two of the strongest association detected. tNAD and NAD^+^ were positively and negatively associated with ATP and PCr, respectively (Figure 3B). ATP and PCr were also negatively correlated. The NAD^+^/NADH redox ratio was positively associated with k_CK_ and k_ATP_, with k_CK_ and k_ATP_ being positively associated, but failed to meet the FDR threshold. ATP and PCr were strongly negatively and positively associated with age, respectively. While none of the NAD variables had a statistically significant association with age, a trend between NAD^+^ and age (negative association *p* = 0.063) and between tNAD and age (negative association *p* = 0.069) were detected. No significant association between NAD^+^/NADH redox ratio and age was observed (*p* = 0.299).

Of the metabolites involved in phospholipid membrane metabolism, the diesters, GPE and GPC, were both negatively associated with NAD^+^ and ATP, and both positively associated with age. GPE was also positively associated with PCr, while PC negatively associated with age.

Mean k_ATP_ was higher in the middle-age group compared to the young group (Figure 2), but we did not find a corresponding significant Spearman's correlation between age and k_ATP_ (Figure 3A). No obvious outlier was observed in the k_ATP_ data set which was normally distributed.

Four metabolite ratios related to energy (PCr/Pi, ATP/PCr, ATP/Pi) and membrane (PME/PDE), and their association with NAD and age were determined (Supplementary Table 4). NAD^+^ was significantly negatively associated with PCr/Pi, while age was negatively associated with ATP/PCr and PME/PDE.

## Discussion

The key findings of our study revealed that brain NAD^+^ and tNAD levels were positively associated with brain ATP level. Moreover, the NAD^+^/NADH redox ratio was positively associated with the two rate constants of ATP energy production, k_ATP_ and k_CK_. NAD was also observed to be part of a metabolic network linking membrane phospholipid metabolism, energy level and aging. In addition, a trend between age and NAD was detected. To the best of our knowledge, this is the first human brain study in which associations were investigated between NAD, ATP energy production and phospholipid membrane metabolism by ^31^P MRS across fifty healthy participants in two different age groups.

### NAD Association With ATP Level and the Influence of Age

Our analysis revealed a positive association between NAD and ATP level. While numerous *in-vitro* and pre-clinical studies have linked NAD and energy homeostasis (Shen et al., 2017; Dienel, 2019; Lautrup et al., 2019; Gilmour et al., 2020; Katsyuba et al., 2020), the current results provide the first direct evidence of an association between ATP and NAD levels in human brain (Figure 4). Mechanistically, this association can be explained based on the dual function of NAD: First, NAD is a key redox cofactor for several enzymes converting glucose, the main source of brain energy, to ATP, the main brain energy currency (Dienel, 2019). During glycolysis, two enzymes dependent on NAD (glyceraldehyde-3-phosphate dehydrogenase and pyruvate decarboxylase) convert 4 NAD^+^ to 4 NADH leading to the formation of 2 acetyl-CoAs that enter the TCA cycle in the mitochondria. Each turn of TCA cycle reduces 6 NAD^+^ to 6 NADH via three enzymes (i.e., isocitrate dehydrogenase, α-ketoglutarate dehydrogenase, malate dehydrogenase). The NADH is then used by the NADH dehydrogenase complex-1 of the ETC to eventually produce ATP.

Second, NAD^+^ is consumed as a co-substrate in non-redox enzymatic reactions involved in energy homeostasis amongst several important roles (Lautrup et al., 2019). In this context, sirtuins, SIRT1 and SIRT3, represent possibly the most sensitive class of enzymes relative to NAD redox ratio and NAD^+^ level (Katsyuba et al., 2020). SIRT1 deacylates and activates transcriptional regulators in the nucleus whereas SIRT3, located in the mitochondria, deacylates and activates multiple metabolic gene targets involved in mitochondrial biogenesis and function. As a result, their activation by NAD^+^ yields beneficial effects on multiple metabolic pathways, including an increase in mitochondrial oxidation rate (Xiao et al., 2018).

We also found that PCr was negatively associated with NAD^+^ and ATP. Supporting this finding, the PCr/Pi ratio was negatively associated with NAD^+^ as well. PCr is considered as a temporal and spatial energy buffer to ATP fluctuation across the brain cell compartments (Schlattner et al., 2006, 2016; Lowe et al., 2013). That PCr is inversely correlated with ATP was previously observed in an aging study (Forester et al., 2010), suggesting that in the middle-aged brain, ATP is not produced or used as efficiently, and hence its relative level compared to PCr is lower than in younger adults. Our study also supports a strong negative association between ATP/PCr ratio and age and between ATP and age, but a positive association between PCr and age. This was also confirmed by the analysis of the middle-age vs. young group that showed a decrease of brain ATP and an increase in PCr levels (Figure 2).

We did not find a strong age-dependent effect on brain NAD content. Comparison of the middle-aged vs. young group showed a non-significant decrease in all NAD parameters. A trend toward a negative association was observed between age and brain tNAD or NAD^+^. The first ^31^P-MRS study to report NAD in the aging human brain found that tNAD, NAD^+^, and the NAD^+^/NADH redox ratio decreased with age in the occipital region (Zhu et al., 2015). Similar findings were found for NAD^+^ measured by ^1^H-MRS (Bagga et al., 2020), but this was not replicated in a recent ^31^P-MRS study in which NAD was not affected by age (Elhassan et al., 2019). When NAD was measured in the frontal lobe of healthy participants (Kim et al., 2017), an age-dependent increase in NADH and decrease in the NAD^+^/NADH redox ratio was observed, but no change in NAD^+^ was detected. Recently it was found that a decrease in blood NAD was only detected in geriatric population (75 to 101 years and hospitalized for decompensated heart failure in a geriatric ward) but no significant correlation between NAD levels and donors' age in the healthy population aged 18 to 68 years (Breton et al., 2020) was found.

One possible explanation for the weak interaction between age and NAD detected in our study is that all the participants were assessed in the fasted state in the morning. Under these controlled diurnal and fasting conditions (not applied in other published studies), it might be that the brain NAD status at rest is less affected by age. Circadian rhythms, which are known to be directly linked with the NAD redox ratio (Lananna and Musiek, 2020), might contribute to a greater age-sensitivity effect on NAD during the day, but less in the fasted or rested conditions. Supporting this view, it was shown that NADH oscillates over time in human red blood cells (O'Neill and Reddy, 2011), and that caloric restriction could restore the circadian rhythm and NAD redox status that is lost in older mice (Sato et al., 2017).

### NAD^+^/NADH Redox Ratio and ATP Metabolic Rate

**Figure 5:**
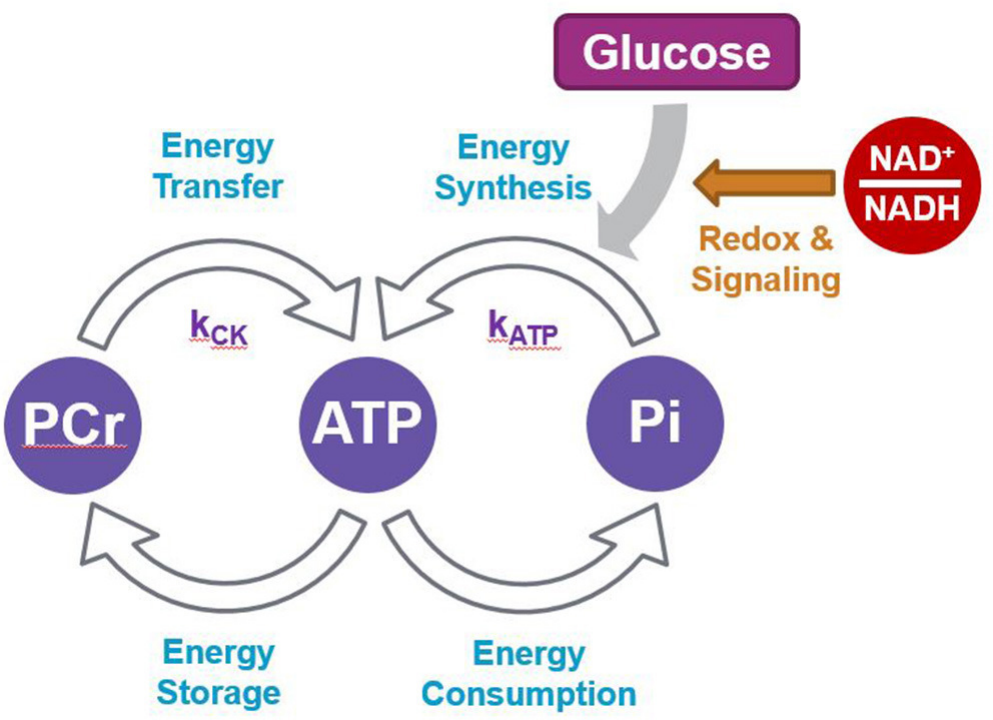
Scheme summarizing the network between ATP and PCr production and utilization.

While it is well-accepted that the NAD^+^/NADH redox ratio is intimately linked with energy metabolism, and that a lower brain ratio is indicative of impaired brain energy regulation (Ying, 2008; Xiao et al., 2018; Lautrup et al., 2019; Katsyuba et al., 2020), direct evidence linking ATP production and NAD in humans has not previously been described in the literature. Here we found that the NAD^+^/NADH redox ratio was positively associated with the rate constants of ATP production, k_ATP_ and k_CK_, and also that k_ATP_ was positively associated with k_CK_. We discussed above how basal ATP and NAD levels are closely associated. Additionally, continuous brain energy supply is tightly regulated by a dynamic exchange system that allows a stable ATP concentration across the cell (Figure 5) (Lei et al., 2003; Zhu and Chen, 2018). Overall, most of brain ATP metabolism is controlled by the activity of ATP synthase which generates ATP from ADP and Pi (k_ATP_) in the mitochondria by oxidative phosphorylation. The energy in mitochondrial ATP is then transferred to PCr via mitochondrial CK. PCr diffuses into the cytoplasm where, under the action of cytosolic isoforms of CK, it generates ATP and Cr (k_CK_). These processes allow energy to be transferred rapidly to various parts of the cell, an energy “shuttle” system particularly important in neurons (Schlattner et al., 2016; Bonvento et al., 2017). Our results suggest that within healthy participants, a more oxidized NAD ratio permits a faster rate of ATP metabolism.

Change in brain ATP metabolic rate has been reported in a few human studies. During visual light stimulation, an increase in both occipital k_ATP_ and k_CK_ were found in healthy young participants, suggesting an increase in ATP flux supported by both CK and ATP synthase to satisfy the increased brain activity (Zhu et al., 2018). Compared to age-matched healthy controls, a first episode of schizophrenia (Du et al., 2014) or bipolar disease (Du et al., 2018) showed both a decrease in prefrontal NAD^+^/NADH (Kim et al., 2017) and k_CK_ without a relative change in ATP/PCr ratio, indicative of a lower resting ATP flux and underlining an energy production failure in these neurological conditions.

Interestingly, we found that k_ATP_ was (+13.9 %) greater in the middle-age group compared to the young group (Figure 2) but did not find a linear correlation between k_ATP_ and aging. This suggests that the relationship between age and k_ATP_ may not be linear, at least not over this age range. The middle-aged group, with a mean age of 56.4 years old and 76% of the subjects between 49 and 59 years old, is at an inflection point for several pre-symptomatic age-related physiological changes. For example, antioxidant defense, evaluated by plasma glutathione/glutathione disulfide redox ratio, is maintained until 45 years old and then declines steadily, indicating a progressive increase of systemic oxidative stress (Jones et al., 2002; Go and Jones, 2017). Brain network stability, a potential biomarker of human brain age, becomes instable from age 47 with the most dramatic change at the age of 60 (Mujica-Parodi et al., 2020). Finally, complexity in neural systems and brain activity, measured as a fractal dimension of EEG signals, follows a bell-curve shape over age, increasing from young to middle-age (50 years old) and then decreasing in older age (Smits et al., 2016). Therefore, our observations that brain NAD redox status is associated with energy production, combined with the relative decrease in ATP level but increased k_ATP_ with age suggest that the healthy but middle-aged brain may compensate for a latent metabolic imbalance by increasing the synthesis rate of ATP to sustain the overall ATP flux at a lower ATP level. Such compensatory mechanism might not operate as effectively in older age (Boumezbeur et al., 2010) and warrants further investigation.

### NAD Is Associated With Phospholipid Membrane Metabolism

Reduced brain volume is one hallmark of brain aging (Resnick et al., 2003), driven by a decrease in neurogenesis and synaptogenesis. Phospholipids are directly implicated in these processes as they provide major structural integrity to the neuropil and myelin. ^31^P-MRS can detect key metabolites of cell membrane phospholipid synthesis [phosphomonoesters (PME): phosphocholine (PC) and phosphoethanolamine (PE)], of membrane phospholipid membrane breakdown (phosphodiesters (PDE): glycerophosphocholine (GPC) and glycerophos-phosphoethanolamine (GPE), and turnover (PME/PDE) (Zhu and Chen, 2018; Downes et al., 2019; Haszto et al., 2020). Hence their quantification provides a measure of membrane expansion and contraction. Previous MRS studies have identified a significant age-related reduction in brain PME (McClure et al., 1995; Blüml et al., 1999; Forester et al., 2010; Schmitz et al., 2018), and an increase in PDE (McClure et al., 1995; Blüml et al., 1999), with two studies reporting a concomitant increase in PCr (Blüml et al., 1999; Forester et al., 2010). Importantly, it has been estimated that phospholipid turnover in the brain consumes a relatively high 13% share of total ATP brain production (Purdon and Rapoport, 1998). Therefore, it is not surprising that correlations have been detected between membrane metabolites, energy molecules, and age.

We found that NAD level was negatively associated with the phosphodiesters, GPC and GPE. GPC and GPE level increased with age and were negatively associated with ATP and positively associated with PCr (Figure 3B). As GPC and GPE represent breakdown products of membrane phospholipids, our results suggest that together with a low NAD and ATP level, the catabolism of brain membrane phospholipids also increases with age. This supports an aging metabolic model in which less energy is available for the maintenance of the cell membranes (Raz and Daugherty, 2018; Cunnane et al., 2020), leading to morphological fluctuations such as myelin and neuropil shrinkage, decrease in brain plasticity, and eventually, impaired information processing capabilities. Mechanistically, the link between NAD and phospholipid metabolism is likely to involve many biological pathways, including Sirtuins and enzymes involved in NAD biosynthesis (Verdin, 2015; Satoh et al., 2017; Lautrup et al., 2019; Sasaki, 2019; Katsyuba et al., 2020).

### Potential Implication for Treatments

Our data are consistent with NAD level and redox state being key players in the efficiency of brain energy metabolism. Therefore, strategies to increase brain NAD and redox ratio should contribute to improve energy metabolism, a major driving factor in brain aging (Cunnane et al., 2020). Importantly, our data show that the age effect on NAD seems to be more heterogenous than anticipated, at least between 19 and 59-year-old, suggesting that some but not all middle-age adults might benefit from an NAD boosting intervention. The ability to modulate brain NAD level in humans has not been demonstrated yet, despite numerous pre-clinical reports showing positive effect of NAD precursor in model of neurological disease (Dienel, 2019; Lautrup et al., 2019; Gilmour et al., 2020). Previously, we have shown that a nutritional ketogenic intervention could increase indirectly the NAD^+^/NADH redox ratio in the brain of healthy young participants (Xin et al., 2018), demonstrating that modulation of NAD in human brain is feasible and can be detected by high field MRS. How this will then contribute to better resilience to the effects of brain aging and cognitive decline remains to be investigated in humans.

## Limitations

Our findings are limited to the occipital region of the brain with mixed tissue composition. Results from other aging studies have shown that the sensitivity to declining glucose metabolism is heterogenous across brain regions (Castellano et al., 2019). Therefore, one could expect a greater effect on energy metabolites and NAD levels in brain areas such as the temporo-parietal-occipital lobes and cingulate gyri while other regions such as the frontal lobes and central structures might be less affected. Similarly, as there are different patterns of age-associated alterations in both gray and white matter volume (Farokhian et al., 2017), investigations in these specific tissues might provide more informative results. Additionally, our study measured global changes in energy level such as ATP and NAD but could not account for changes in specific cellular compartments, e.g., cytosol/glycolysis vs. mitochondria/oxidative phosphorylation, or different brain cell types.

Our study did not include many participants over 65 years old. This limited our interpretation to a middle-aged population and precluded robust extrapolation to older age, where more pronounced energy deficit might be observed. Moreover, all participants were investigated in a fasting state at rest. It will be important to determine how circadian rhythms impact brain phosphometabolite levels and the rate of ATP synthesis, and whether aging amplifies these changes. While many correlations withstood the FDR correction for multiplicity analysis, conclusions related to energy metabolic rate remain tentative, in part due to their higher variability.

Therefore, additional studies involving several brain regions under various diurnal and metabolic status in a wider age population will be required to provide a more definitive answer as to how much, where and when human brain NAD redox status is most affected by age.

## Conclusion

Overall, our results support the hypothesis that an optimum brain NAD^+^/NADH redox ratio and NAD level sustains high ATP production and levels in the brain. This implicates more efficient glucose metabolism all the way from the first steps of glycolysis to ATP production by oxidative phosphorylation in the mitochondria. It also highlights the possible implication of NAD in a metabolic network linking membrane phospholipid turnover with energy production and aging.

Our study demonstrates also that the direct determination of brain ATP metabolic rate is a relevant and sensitive physiological parameter that helps characterize brain aging. It will provide an important and complementary approach to the well-established metabolic rate measures of various brain energy substrates such as glucose, oxygen, lactate or ketones.

Efficient regulation of intracellular ATP homeostasis by dynamic adjustment of the ATP metabolic fluxes to effectively balance mitochondrial ATP production and utilization appear to be a general phenomenon in the healthy human brain. Therefore, maintaining ATP level and flux via optimization of energy substrates and NAD levels may be a promising therapeutic strategy aimed at preserving brain health and preventing cognitive decline with age, the main risk factor for neurodegenerative diseases.

## Data Availability Statement

The studies in young and middle-age participants were approved by the Ethics Committee of Canton de Vaud (Switzerland) under the references 2017-00159 and 2018-00463, respectively, and all participants provided written informed consent.

## Ethics Statement

The studies involving human participants were reviewed and approved by Studies in young and middle-age participants were approved by the Ethics Committee of Canton de Vaud (Switzerland) under the references 2017-00159 and 2018-00463, respectively, and all participants provided written informed consent. Procedures were conducted according to the principles of the Declaration of Helsinki. Trial registration numbers for studies in young and middle-age participants were NCT03101345 and NCT03541473, respectively. The patients/participants provided their written informed consent to participate in this study.

## Author Contributions

LX, ÖI, RG, MB, MS, SC, and BC designed the studies. Data collection was performed by LX and ÖI. MS, LX, and BC analyzed data. The manuscript was drafted by BC and LX. All authors discussed the results and revised the manuscript.

## Conflict of Interest

BC, MS, and MB were employees of Nestle when this study was conducted. SC had done consulting for/or received honoraria from Bulletproof, Keto-Products, Accera/Cerecin, Nestlé Health Science, Nisshin Oillio, and Pruvit. Nestlé Health Science had funded some research by SC's group. SC had recently formed a company, SENOTEC Inc., to develop ketogenic products. The authors declare that this study received funding from Nestle. The funder had the following involvement in the study: design of the studies, data analysis, drafting and revising the manuscript. The remaining authors declare that the research was conducted in the absence of any commercial or financial relationships that could be construed as a potential conflict of interest.
