# Supplementary material for: Brain NAD Is Associated With ATP Energy Production and Membrane Phospholipid Turnover in Humans
Source: Front Aging Neurosci. 2020 Dec 16;12:609517. doi: 10.3389/fnagi.2020.609517 (PMC7772416; doi:10.3389/fnagi.2020.609517)
Supplement: Supplementary file 1 [file Data_Sheet_1.pdf]

Supplementary Material

Brain NAD is associated with ATP energy production and membrane phospholipid turnover in humans

Bernard Cuenoud[1]*, Özlem Ipek[2], Maya Shevlyakova[3], Maurice Beaumont[3], Stephen C. Cunnane[4], Rolf Gruetter[5], Lijing Xin[6]*

*** Correspondence**:

Bernard Cuenoud, bernard.cuenoud@nestle.com

Lijing Xin, lijing.xin@epfl.ch

Table S1: Detailed participant demographic data in the young (A) and middle-age (B) groups. For group B, 28 subjects were enrolled in the study. Three participants dropped out before completion and were not included in the analysis.

(A)

	Female					Male				
	N	Mean	SD	Min	Max	N	Mean	SD	Min	Max
Age (years)	8	25.5	2.5	22.0	30.0	17	27.2	7.1	19.0	38.0
Height (m)	8	1.65	0.08	1.56	1.78	17	1.79	0.06	1.70	1.93
Weight (kg)	8	58.56	8.65	47.0	73.0	17	72.94	7.79	60.0	93.0
BMI (kg/m^2)	8	21.32	1.94	19.13	24.41	17	22.70	1.48	18.94	24.97

(B)

	Female					Male				
	N	Mean	SD	Min	Max	N	Mean	SD	Min	Max
Age (years)	13	55.6	4.8	51.5	64.9	15	57.0	7.60	50.3	72.0
Height (m)	13	1.68	0.10	1.53	1.8	15	1.79	0.07	1.69	1.93
Weight (kg)	13	65.7	9.29	53.0	82.0	15	77.8	7.79	62.0	90.0
BMI (kg/m^2)	13	23.34	2.69	18.56	27.18	15	24.40	2.33	20.96	29.73

Figure S1: Participants age distribution

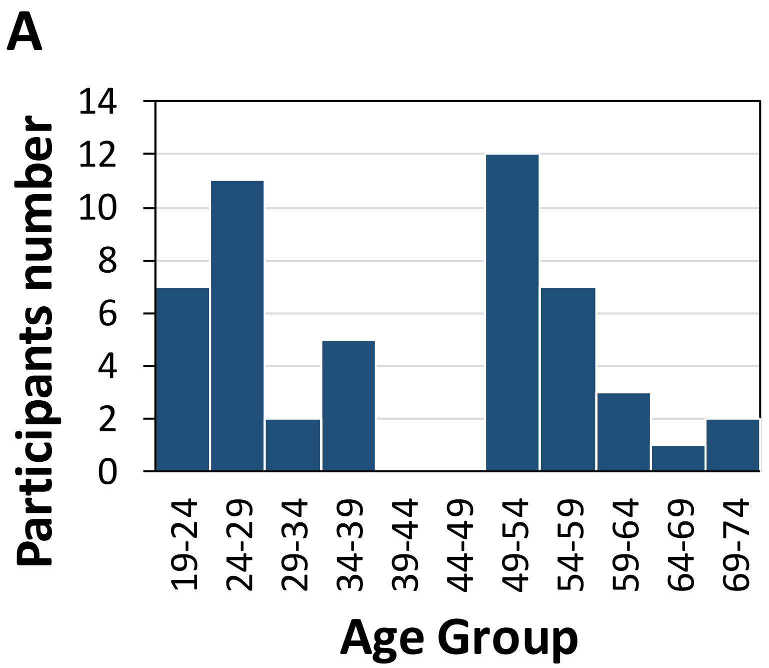

Table S2: Percent change in phospho-metabolites and in metabolic rate constants between the middle-age (MA) group versus the Young group. Average values for metabolite levels are reported as percent total phosphometabolites measured, and as $s^{-1}$for metabolic rates; SD Standard Deviation. Percent changes are ranked according to their p-values. Raw p-values that pass the FDR correction are in bold.

	Average MA	SD	Average Young	SD	% change MA vs Young	p
GPE	3.86	0.28	3.32	0.24	16.2	**2.42E-09**
ATP	17.25	0.38	17.78	0.31	-3.0	**2.25E-06**
PCr	17.91	1.03	16.96	0.92	5.6	**1.29E-03**
GPC	5.57	0.47	5.22	0.48	6.7	**0.0120**
kATP	0.15	0.02	0.13	0.03	13.9	0.022
PE	7.32	0.39	7.56	0.34	-3.1	0.030
tNAD	1.49	0.09	1.54	0.11	-2.9	0.12
NAD+	1.27	0.09	1.31	0.10	-3.1	0.15
Pi	5.13	0.27	5.04	0.32	1.8	0.28
RX	5.78	1.34	6.08	1.81	-4.9	0.51
kCK	0.23	0.03	0.23	0.02	-1.1	0.71
NADH	0.23	0.04	0.23	0.07	-1.9	0.78
PC	3.52	0.37	3.52	0.44	0.0	0.99

Table S3: Spearman's coefficient (Rho value) list with uncorrected p-values below 0.05. Rho values are ranked according to their p-values. Raw p-values that pass the FDR correction are in bold.

	Correlation	Spearman's Rho	p
1	**Rx vs NADH**	**-0.942**	**1.50E-24**
2	**tNAD vs NAD+**	**0.838**	**3.13E-14**
3	**GPE vs Age**	**0.733**	**1.39E-09**
4	**ATP vs PCr**	**-0.649**	**3.31E-07**
5	**ATP vs GPE**	**-0.639**	**5.85E-07**
6	**ATP vs Age**	**-0.592**	**5.82E-06**
7	**GPE vs GPC**	**0.559**	**2.35E-05**
8	**PCr vs GPE**	**0.524**	**9.18E-05**
9	**ATP vs tNAD**	**0.467**	**6.15E-04**
10	**ATP vs GPC**	**-0.448**	**0.00109**
11	**ATP vs NAD+**	**0.442**	**0.00130**
12	**Rx vs NAD+**	**0.44**	**0.00138**
13	**PCr vs Age**	**0.433**	**0.00182**
14	**GPC vs Age**	**0.427**	**0.00198**
15	**PE vs Age**	**-0.379**	**0.00660**
16	**PCr vs NAD+**	-0.366	0.00883
17	**NAD+ vs GPE**	-0.35	0.0124
18	**tNAD vs NADH**	0.35	0.0124
19	**tNAD vs PCr**	-0.34	0.0153
20	**kATP vs kCK**	0.324	0.0258
21	**GPE vs tNAD**	-0.305	0.0310
22	**GPC vs tNAD**	-0.298	0.0353
23	**Rx vs kCK**	0.304	0.0375
24	**NAD+ vs GPC**	-0.294	0.0380
25	**Rx vs kATP**	0.302	0.0385
26	**kCK vs PCr**	-0.288	0.0492

Table S4: Spearman correlations between metabolite ratios, NAD and aging. Bold numbers indicated significant correlation ($p < 0.05$). No FDR correction was applied to this separate analysis.

**Spearman**	**NADH**	**NAD+**	**tNAD**	**Rx**	**Age**
**PCr/Pi**	0.215	**-0.301**	-0.160	-0.225	0.249
**ATP/PCr**	-0.118	0.199	0.120	0.102	**-0.559**
**ATP/Pi**	0.125	-0.163	-0.082	-0.154	-0.274
**PME/PDE**	0.059	0.208	0.230	0.053	**-0.584**
